# Kinetics of Organic Biodegradation and Biogas Production in the Pilot-Scale Moving Bed Biofilm Reactor (MBBR) for Piggery Wastewater Treatment

**DOI:** 10.1155/2021/6641796

**Published:** 2021-01-05

**Authors:** Thi Ha Nguyen, Manh Khai Nguyen, Thi Hoang Oanh Le, Thanh Tu Bui, Trong Hieu Nguyen, Truong Quan Nguyen, Anh van Ngo

**Affiliations:** ^1^Faculty of Environmental Sciences, VNU University of Science, Vietnam National University, Hanoi, Vietnam; ^2^Faculty of Mathematics, Mechanics and Informatics, VNU University of Science, Vietnam National University, Hanoi, Vietnam; ^3^Research Centre for Environmental Technology & Sustainable Development, VNU University of Science, Vietnam National University, Hanoi, Vietnam

## Abstract

In this research, the kinetics of COD biodegradation and biogas production in a moving bed biofilm reactor (MBBR) at pilot scale (10 m^3^) for piggery wastewater treatment were investigated. Polyethylene (PE) was used as a carrying material, with organic loading rates (OLRs) of 10, 15, and 18 kgCOD/m^3^ day in accordance to hydraulic retention times (HRTs) of 0.56, 0.37, and 0.3 day. The results showed that a high COD removal efficiency was obtained in the range of 68–78% with the influent COD of 5.2–5.8 g/L at all 3 HRTs. About COD degradation kinetics, in comparison to the first- and second-order kinetics and the Monod model, Stover–Kincannon model showed the best fit with *R*^2^ 0.98 and a saturation value constant (*K*_*B*_) and a maximum utilization rate (*U*_max_) of 52.40 g/L day and 82.65 g/L day, respectively. The first- and second-order kinetics with all 3 HRTs and Monod model with the HRT of 0.56 day also obtained high *R*^2^ values. Therefore, these kinetics and models can be further considered to be used for predicting the kinetic characteristics of the MBBR system in piggery wastewater treatment process. The result of a 6-month operation of the MBBR was that biogas production was mostly in the operating period of days 17 to 80, around 0.2 to 0.3 and 0.15–0.20 L/gCOD_converted_, respectively, and then reduction at an OLR of 18 kgCOD/m^3^. After the start-up stage, day 35 biogas cumulative volume fluctuated from 20 to 30 m^3^/day and reached approximately 3500 m^3^ for 178 days during the whole digestive process. Methane is accounted for about 65–70% of biogas with concentration around 400 mg/L.

## 1. Introduction

A major environmental problem that relates to piggery wastewater is the lack of appropriate wastewater treatment technologies, especially in developing countries. Moving bed biofilm reactor (MBBR) is an improved technique of biological process used for wastewater treatment. MBBR system design is made of conventional activated sludge process combined with biofilm media. MBBR system includes an activated sludge aeration system in which the sludge is collected on plastic carriers [1]. In order to optimize the contact of water, air, and bacteria, these carries must have a large internal surface. The bacteria/activated sludge will then grow on the carrier's internal surface and decompose the organic matters in the piggery wastewater.

Previous research studies showed that anaerobic digestion provides potential benefits of methane production together with waste management [2]. The high loading rate of anaerobic reactors is preferred to be used worldwide because they are designed to operate at short HRTs and long SRTs to incorporate a large amount of high active biomass; hence higher loading capacity and improved sludge stabilization are allowed [3]. The MBBR system consists of a biological treatment process based on the microbial adhesion mechanism in activated sludge and microbial material system that moves between the two layers to create a microbial wastewater treatment membrane. In recent years, there are many research studies focusing on the MBBR system with new modifications in carrier materials. These carriers are usually made of various materials such as porous materials and recycled plastic and have a large surface inside to contact with water, air, and bacteria in an optimal way. The research results of Borkar et al. showed that polyethylene (PE) is a biofilm carrier that may have great potential to be used in the MBBR to remove organic matter from water and wastewater [4]. That is why PE carrier was used in this study.

To have a better understanding about kinetics in the digestion process, mathematical models have been developed and applied. These are very effective tools to learn well about the mechanism of biotransformation and degradation of compositions within a digester [5]. Obviously, a mathematical model is an essential tool to observe, predict, simulate, and optimize the system's kinetics or mechanism at different operations. In anaerobic digestion, the kinetic models were developed concerning the type and characteristic of substrates. The models simulated the substrate degradation kinetics, intermediate (e.g., VFAs) production, and biogas production [6]. The kinetic of biodegradation is studied with some key quantities including microbial growth rate, substrate utilization rate, biokinetic coefficients, and growth constants. The performance of the system (biomass production or substrate degradation) depends on the substrate or nutrient contents and reactor environment (pH, temperature, and dissolved oxygen) [7].

The first- and second-order equations have been used to simply simulate the biodegradation mechanism during anaerobic digestion process [8]. In addition, among the number of models being applied for substrate decomposition and methane production kinetics, Monod and Stover–Kincannon models seem to be widely used [9–12].

The objective of this study is to investigate the kinetics of organic biodegradation and methane production during piggery wastewater treatment process using the MBBR at pilot scale. The findings will provide additional information to consider and select appropriate solutions and optimal operation conditions.

## 2. Experimental Methods

### 2.1. Anaerobic MBBR System

**Figure 1:**
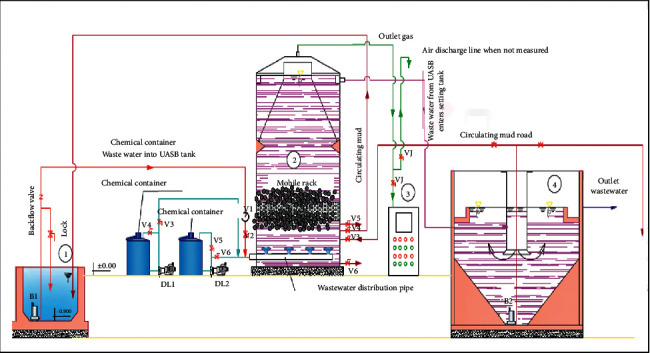
Schematic diagram of the pilot-scale anaerobic MBBR system.

A schematic diagram of the pilot-scale anaerobic MBBR system is shown in Figure 1.

The MBBR system was designed based on the results of Nguyen et al. in terms of influent COD ranging 5.0–6.0 g/L; PE carrier; and temperature [13] in the lab-scale MBBR, including (1) piggery wastewater (equalization tank): 20 m^3^ (L × W × D: 5 × 4 × 1 m); (2) cylinder anaerobic reactor (D × H: 2.4 × 3.6 m) with working volume 10 m^3^; (3) SSM 6000 LT Pronova-Gas Analysers-multichannel measuring device, Germany; and (4) settling tank: 5 m^3^. There are 2 chemical containers (acid and base) to adjust influent pH. However, in this study, pH of influent wastewater does not need to be adjusted.

Biofilm carrier is a PE wheel-shaped material (D × H: 15 × 10 mm) with a specific surface area of 800–1000 m^2^/m^3^ and density of 60 kg/m^3^, filled about 30% of MBBR volume.

The seed anaerobic sludge was collected from the upflow anaerobic sludge blanket (UASB) tank of the wastewater treatment plant (capacity: 600 m^3^/day) in the Sabeco Beer Manufacturing Plant. The start-up (lag phase) lasts for 12 days and continues to operate with an OLR of 5 kgCOD/m^3^ day until day 34 before OLR was increased to the ranges of 10–18 kg COD/m^3^ day. Initial sludge concentration was 3.5–4.0 kg/m^3^; after 35 days, the sludge concentration was increased to 5.5 to 6.0 kg/m^3^.

The influent of piggery wastewater passed the screening (5 × 10 mm) into the equalization tank and was then pumped to an anaerobic MBBR tank with flow rates of 18, 27, and 33 m^3^/day for the OLRs of 10, 15, and 18 kgCOD/m^3^ day, respectively.

### 2.2. Sampling and Analytical Methods

Influent and effluent wastewater samples (48 samples each) were taken twice a week during an approximately 6-month operation from day 35. Sampling methods follow Vietnamese standards TCVN 5992-1995, and TCVN 5993-1995 about water quality-sampling-guidance on sampling techniques; pH was measured online by 4801P pH/ORP Controller, Gondo, Taiwan (triplicate). COD_*o*_ and COD_*t*_ values that were used in kinetic models were calculated as average values for each HRT and OLR. COD, TSS, TN, and TP parameters were analyzed by TCVN 6491 : 1999, TCVN 6625 : 2000, TCVN 6638 : 2000, and TCVN 6202 : 2008, respectively.

Biogas production and methane concentration were measured by SSM 6000 LT Pronova-Gas Analysers-multichannel measuring device, Germany. Data measurement occurs twice a day at 8-9 am and 3-4 pm. Error of methane volume measurement is ±1%. To ensure accurate gas measurement results, sensors are automatically calibrated using the Pronova proCal mode for CH_4_ and CO_2_ and periodic calibration for H_2_ and H_2_S gases. In addition, calibration was also performed by 2 measuring points using reference gases. Methane concentration in biogas was also analyzed by GC according to TCVN 8715-2011.

### 2.3. Applied Kinetics Equations

COD degradation was considered in applications of the following kinetics.

First-order kinetics:

(1) $$\begin{array}{c} \text{Ln}\left(\frac{{\text{COD}}_{t}}{{\text{COD}}_{o}}\right)=-k_{1}t+b. \end{array}$$

Second-order kinetics:

(2) $$\begin{array}{c} \frac{1}{{\text{COD}}_{t}}=k_{2}t+b. \end{array}$$

#### 2.3.1. Monod Model

Monod kinetic model is when given a complete mixing system, it is able to find out the substrate utilization rate linked to the particular growth rate [14]. The rate of change in substrate concentration is insignificant at stable state conditions; the Monod equation can be given in the reparametrized form:

(3) $$\begin{array}{c} -\;r_{\text{su}}=\frac{\left({\text{COD}}_{o}-{\text{COD}}_{t}\right)}{\text{HRT}}=k_{1}\cdot {\text{COD}}_{t}, \end{array}$$

where *r*_su_ is the substrate utilization rate (g/m^3^ day), COD_*o*_ is the concentration of the limiting substrate for growth (g/m^3^), COD_*t*_ is the rest of the substrate concentration (g/m^3^), HRT is the hydraulic residence time (day), *k*_1_ is the first-order rate constant (1/day), and *k*_2_ is the second-order rate constant (m^3^/g day).

The slope *k*_1_ can be obtained by plotting ((COD_*o*_ − COD_*t*_)/HRT) versus COD_*t*_ in equation (3). First-order reaction and Monod degradation model were also applied for biodegradation kinetics of synthetic high COD wastewater using microalgal species *Chlorella pyrenoidosa* [10].

#### 2.3.2. Stover–Kincannon Model

It was developed to design concepts describing total OLR and establishing a kinetic model for the biofilm reactor. Stover–Kincannon model is found to be one of the best mathematical models for the substrate removal rate's demonstration. This equation was used to compute the reactor volume and effluent organic concentration for the reactor operating in stable state conditions. Substrate utilization rate is only considered as a function of the OLR as shown in the following equations:

(4) $$\begin{array}{c} \frac{V}{Q\left({\text{COD}}_{o}-{\text{COD}}_{t}\right)}=\frac{K_{B}}{U_{\max}}\left(\frac{V}{Q{\text{COD}}_{o}}\right)+\frac{1}{U_{\max}}, \end{array}$$

(5) $$\begin{array}{c} \frac{\text{HRT}}{\left({\text{COD}}_{o}-{\text{COD}}_{t}\right)}=\frac{K_{B}}{U_{\max}}\left(\frac{\text{HRT}}{{\text{COD}}_{o}}\right)+\frac{1}{U_{\max}}, \end{array}$$

where *K*_*B*_ is the saturation value constant and *U*_max_ is the maximum utilization rate.

The saturation value suggests that the substrate has been removed by microorganisms, and the maximum utilization rate shows that the maximum substrate has been removed by aerobic organisms versus time. When plotting (HRT/(COD_*o*_ − COD_*t*_)) vs. (HRT/COD_*o*_), the linear relationship will be obtained as the first-order kinetic for *U*_max_ and *K*_*B*_ identification.

## 3. Results and Discussion

### 3.1. Characteristic of Influent Piggery Wastewater

The results of 48 sampling surveys which show the characteristics of piggery wastewater used as influent wastewater for the pilot-scale anaerobic MBBR system are summarized in Table 1.

It can be seen that except for pH, all key parameters in influent piggery wastewater do not meet the effluent standards. In particular, very high organic content with COD and BOD values exceeds 50–60 times in comparison with the standard regulated in QCVN01-79 : 2011/BNNPTNT, column B, and about 20 times in comparison with QCVN 62 : 2016/BTNMT, column B. TSS, TN, and TP contents were also found to be very high which, respectively, were 13–25, 3–20, and 10–13 times greater than those in regulated standards.

#### 3.1.1. pH Variation

**Figure 2:**
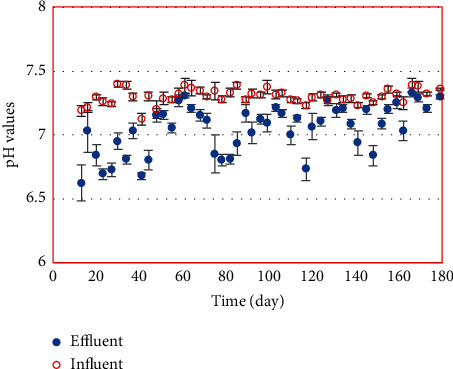
pH variation during operation of the MBBR system (*n* = 3).

**Figure 3:**
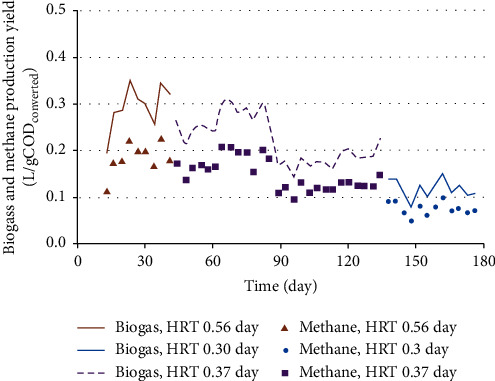
Biogas and methane production yield at different HRTs.

pH is considered to be one of the most sensitive environmental parameters in the anaerobic process. The stability and buffering capacity of the reactor are reflected by the pH value of wastewater in the reactor. The pH variation of influent and effluent piggery wastewater of the pilot-scale MBBR during operation showed that pH values varied within the optimal neutral pH range for bacteria growth (Figure 2). In addition, the pH values of effluents found to be a little lower than those of influents. The reason may be because of the formation of VFAs during degradation process, and not all VFAs were converted to methane. As can be seen in Figure 3, the highest biogas and methane production yields reached 0.35 and 0.22 L/gCOD_converted_, respectively.

The pH values are in the range as recommended for the healthy environment of methane forming bacteria in the digester (6.8–7.4) [15, 16]. However, in practice, the digestion process can work with a pH range of 6.5–8.0 [17]. These pH ranges also minimize the toxicity of both free ammonia and free-volatile fatty acids [18].

In the study of Sun et al., low pH (around 5.0) caused the inhibition effect on the methanogenic biomass during anaerobic digestion. It was observed that, at pH 5.1, the specific decomposition rate increased about 10 times compared to pH 7.0 [19]. However, in this study, influent pH did not adjust to acidic level to ensure appropriate effluent pH ranging from 6.5 to 7.5 for the next treatment stage.

### 3.2. COD Removal and Biodegradation Kinetics

#### 3.2.1. COD Removal Efficiency

**Figure 4:**
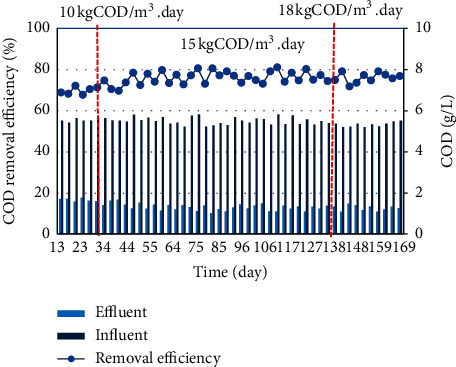
COD removal efficiency at different OLRs.

Figure 4 shows that, with OLRs of 10, 15, and 18 kgCOD/m^3^ day, the influent COD varied in the range of 5.2–5.8 g/L and was rather stable because of the equalization tank. The effluent COD values ranged from 1.1 to 1.8 g/L. The COD removal efficiency obtained was relatively high, mostly in the range of 66–78%. However, effluent wastewater has not yet met the permitted standard, and further treatment steps should be applied. The findings are not in agreement with the results of Esmaeilirad et al., where COD removal efficiency of the pilot-scale MBBR (30 m^3^) clearly depended on the HRT and influent COD. The COD removal efficiencies increased from 65–80 to nearly 90% in accordance with HRT 10 and 48 hrs with COD ranged from 550 to 1500 mg/L [9]. The organic degradation efficacy with respect to TOC values was significantly affected by influent concentrations as 90 and 38% reduction in TOC were obtained for 1000 and 5000 mg/L influent COD wastewater, respectively [10]. However, in small lab-scale anaerobic packed column reactor (6 liters), the COD removal efficiency was found very low which ranged between 5 and 35% for the applied OLRs 1–8 g/L day [20].

At the laboratory scale, Chiemchaisri et al. used an integrated reverse flow system combined with 1.5 × 1.5 × 1.5 cm^3^ porous material (UAFF-upflow anaerobic floating filter) to create a microorganism film for COD and suspended solid removal in anaerobic conditions for pig sewage treatment [21]. The higher COD removal performance reached 89% probably due to the lower OLRs, ranging from 4.2 to 6.1 gCOD/L day.

According to research by Sombatsompop et al. [22], comparing the SBR system with the MBBR using porous bearing material to treat pig wastewater at a low organic load of 0.59–2.36 kgCOD/m^3^ day, COD removal efficiency of both systems at the load from 1.18 to 2.36 kgCOD/m^3^ day reached over 80%. As the load increases, the MBBR system provides better processing efficiency and stability than the SBR system.

#### 3.2.2. First- and Second-Order Kinetics

The COD degradation rates were determined by applying first- and second-order kinetics and Monod and Stover–Kincannon models. Based on equations (1)–(3) and (5), the relationship between influent COD values (COD_*o*_) and effluent COD values (COD_*t*_) of the pilot-scale anaerobic MBBR was investigated during continuous digestion time to identify which model is the most appropriate for the experimental data.

**Figure 5:**
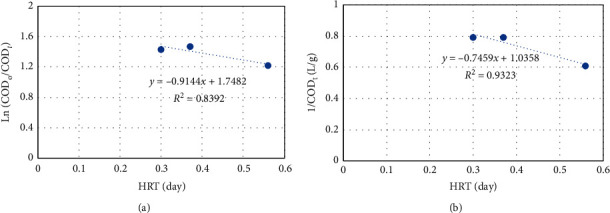
First-order (a) and second-order (b) kinetics of COD degradation in the pilot-scale MBBR.

According to Figure 5, the COD biodegradation seemed to match with the first- and second-order kinetics with high regression coefficients (*R*^2^ values) of 0.84 and 0.93, respectively. These results were similar to some previous studies which found *R*^2^ values ranging from 0.75 to 0.98 [8, 23]. The *k*_1_ and *k*_2_ values were found to be 0.9144/day and 0.7459 L/g day. In comparison with previous studies where the data were collected within different operation times, 20–30 days [8]; 60–80 days [9, 24]; 120 days [25], there was no significant difference of first- and second-order rate constants.

Laowansiri et al. studied the kinetics of chicken slaughterhouse wastewater treatment in the UASB system with COD contents of 400, 800, 1200, and 1600 mg/L at pH 7.00 ± 0.02. The results showed that the reaction order in the degradation of chicken slaughterhouse wastewater having higher COD contents ranging from 800 to 1600 mg/L matches the first-order kinetic model, whereas the second-order kinetic was a better fit at a low COD (400 mg/L). It was also found that the biogas production yield increased with an increase in COD contents and HRT. The highest biogas production reached 267.0 mL biogas volume (64.03% CH_4_) with wastewater treatment of COD concentration at 1600 mg/L for 30 days [8].

#### 3.2.3. Monod Model

**Figure 6:**
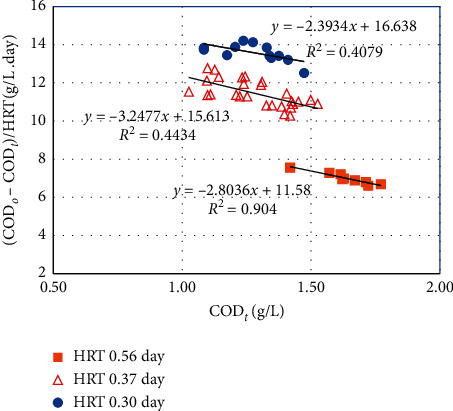
Monod model plot of COD removal in the pilot-scale MBBR at different HRTs.

As in equation (3), the *k*_1_ value was determined from the slope of the line plotting (COD_*o*_ − COD_*t*_)/HRT versus COD_*t*_. From Figure 6, the *k*_1_ values of 2.804, 3.248, and 2.393/day were obtained for HRTs of 0.56, 0.37, and 0.3 day, respectively. However, the exception is HRT 0.56 day where regression coefficient (*R*^2^) obtained was 0.90; shorter HRTs resulted in lower *R*^2^ values approximately 0.40–0.44, which indicates that the experimental data did not fit well with the first-order kinetics. This finding was in agreement with the study of Esmaeilirad et al. where the *k*_1_ value of 3.463/day was determined with a correlation coefficient of 0.41 [9]. The negative mark of *k*_1_ in all 3 equations indicates that when COD_*t*_ increases, the value of (COD_*o*_ − COD_*t*_)/HRT decreases due to COD_*o*_ and HRT being considered constant.

Abu-Reesh investigated the anaerobic digestion of labaneh whey in a 100 L batch reactor and monitored the biogas production versus chemical oxygen demand (COD) content with time. Four kinetic models of Monod, logistic, Contois, and Tessier were studied in comparison with the model predictions and experimental data for COD contents. The findings showed that experimental data fitted all four models of which the Tessier model was found to fit a little better than other tested models [26].

In a partially packed upflow anaerobic fixed film (UAF) reactor with synthetic rubber wastewater having COD of 6355–6735 mg/L and batch operation at five HRTs of 17, 14, 10, 8, and 5 days, the experimental data were analyzed using the Monod model, the modified Stover–Kincannon model, and the Grau second-order model. The result indicates that the data had the greatest match with the Grau second-order model [12]. By applying the data in a Monod kinetic model, it is able to obtain the kinetic parameters for pentachlorophenol (PCP) and 2,4,6 trichlorophenol. The model was capable of projecting simultaneous multisubstrate degradation of PCP with other CPs [11].

#### 3.2.4. Stover–Kincannon Model

**Figure 7:**
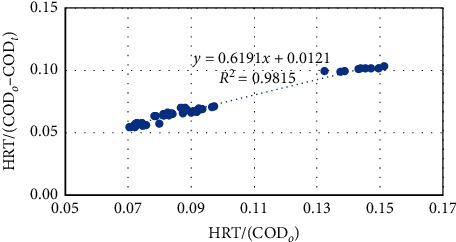
Stover–Kincannon model plot for COD removal in the pilot-scale MBBR.

This model was originally developed by Borghei [27] for the MBBR and was applied for the partially packed upflow anaerobic fixed film reactor treating low-strength synthetic rubber wastewater [12]. The equation was used to study the relationship between specific substrate removal rates and the organic loading rate [28]. The application of Stover–Kincannon model (Figure 7) showed the correlation of HRT/(COD_*o*_ − COD_*t*_) vs. HRT/COD_*o*_. As can be seen from the equation of the linear relationship, the saturation value constant (*K*_*B*_) and maximum utilization rate (*U*_max_) in Stover–Kincannon model were determined as 52.40 g/L day and 82.65 g/L day. The *R*^2^ value was found to be about 0.98 indicating that the experimental data quite match with this model. The findings agreed with the study of Esmaeilirad et al. [9] and Kapdan [20]. However, in their studies, *K*_*B*_ (12.32 and 37.9 g/L day) and *U*_max_ (11.74 and 12.9 g/L day) were much lower, whereas the *R*^2^ value was very high and similar (0.99 and 0.97).

The higher efficiency of the substrate removed by microorganisms (representative by *K*_*B*_) and the maximum substrate removed by aerobic organisms according to time (representative by *U*_max_) may be because of the differences in aerobic and anaerobic degradation conditions and operation times of 60 and 178 days, respectively, of Esmaeilirad et al.'s study [9] and this study. Stover–Kincannon model was applied for the bench-scale UAF reactor to predict the process. As reported, the constants *K*_*B*_ and *U*_max_ of 6.57 and 6.31 g/L day were obtained, respectively [12].

Based on the calculated kinetic coefficients and regression coefficients, Stover–Kincannon model and Monod model with the HRT of 0.56 day better fitted than the first-order model and Monod model with 2 short HRTs to predict the performance of the pilot-scale MBBR (Table 2).

### 3.3. Biogas and Methane Production

Figure 3 shows the biogas production yield of the pilot-scale MBBR at 3 HRTs. It can be seen that the biogas production decreased with the reduction of HRTs from 0.56 to 0.3 day which was in accordance with the increase of OLRs from 10 to 18 kgCOD/m^3^ day. During the first 80 days, the MBBR system stably operated; the biogas yields were found to be mostly around 0.2 to 0.3 L/g COD_converted_. Sometimes, the biogas production yield reached its peak of 0.34–0.35 L/g COD_converted_, while methane yield attained majority in the range of 0.15–0.23 L/g COD_converted_. If the COD/VS ratio was assumed from 1.2 to 1.6 as being reported in the studies of Bullock et al. [29] and Hallaji et al. [30], these results are in agreement with Yiang et al. [28]. In Yiang et al.'s study, methane production yield was found to be 0.263 L/g VS in dry codigestion systems feeding food waste/pig manure (1 : 1) without pH adjustment. In Laowansiri et al.'s study [8], with 1600 mg/L COD in wastewater and a 30-day operation, the highest biogas production obtained was 0.267 L/g COD, and the analyzed methane content was 64.03%.

The biogas production yield was found to be lower than the reported data of previous studies [31, 32]. This was probably due to the difference of the influent substrate. In the study of Hallaji et al. [30], food waste and cow slurry were used for laboratory-scale batch anaerobic digesters. Higher biogas production yields were obtained with food waste, 0.435 L CH_4_/g VS_fed_. The amount of biogas yield also depends on other substrates and processes. The maximum biogas production yield reached 0.61–0.93 L/g VS with energy crops and animal waste slurry feed [33].

Kinetics of biogas production in the anaerobic digestion single-stage reactor of food waste were investigated using the ﬁrst-order kinetic and the modiﬁed Gompertz and the logistic function models. It was found that, among the three models, the modiﬁed Gompertz model was the best ﬁt with the experimental data. The result of the study on the influence of HRTs ranging from 35 to 124 days showed that HRTs played an important role in controlling the stability and performance of degradation process. HRTs clearly affected the intermediate metabolism, biogas production rate, methane yield, and removal eﬃciency [34]. However, in the study of Hassan and Nelson [35], other factors such as types of microorganisms, feed C : N ratio, HRT, reactor design, temperature, pH control, hydrogen pressure, and additives were investigated in order to assess the way they affected the efficiency and stability of the anaerobic digestion process.

**Figure 8:**
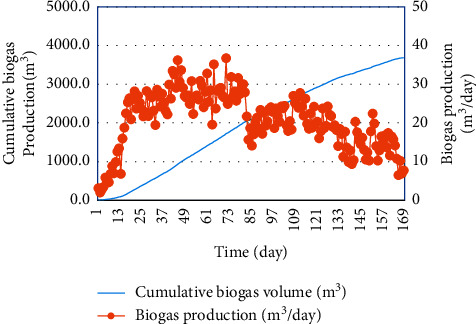
Biogas production and cumulative curve during anaerobic digestion in the pilot-scale MBBR.

Daily measurement of biogas production and cumulative biogas volume is shown in Figure 8. It was found that, during the first 10 days, the biogas kept rising and reached about 10 m^3^/day. After that, it significantly increased up to 22 m^3^/day on the 17^th^ day. Between the 18^th^ day to 80^th^ day, the biogas volume produced per day fluctuated in the range of 20–30 m^3^/day, and some days, it even reached a peak of around 35 m^3^/day. Then, the biogas production seemed to reduce, especially from the 150^th^ day. The cumulative biogas volume gradually gained and attained approximately 3500 m^3^ during the whole digestive process of 178 days.

As shown in Figure 8 for the change of biogas production over time, the impact of accepting input OLRs may be due to the temperature difference between summer and winter. The MBBR system started operating in June 2019, so the temperature in the first 70–80 days is at least 5–15°C higher than that in the later period (autumn and winter). Therefore, the efficiency of decomposition and biogas production were higher. In addition, due to the new piglet litter in June (the average piglet weight is 10–12 kg) and the export in December (the weight is about 100–120 kg), it was possible that the input wastewater composition may be disturbed. During the farming process, according to the age of pigs (farming time), different food and farming care conditions were used. Unfortunately, these components were not thoroughly understood in this research.

**Figure 9:**
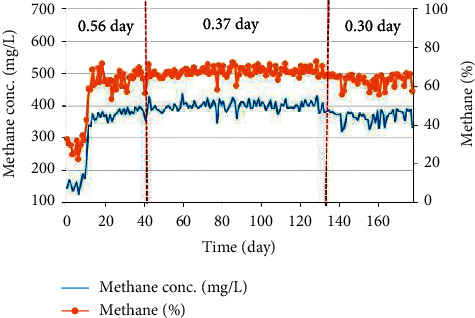
Methane content in biogas produced from the pilot-scale MBBR.

The percentage of methane in biogas mostly ranged from 65 to 70% when the MBBR stably operated from the 15^th^ day till the 120^th^ day. Since then, the percentage of CH_4_ content had decreased to 60–67%. At the same time, the results of biogas production yields were also corresponding to the decrease in the COD value in effluents at different OLRs (Figure 9). This was in agreement with Maragkaki [33] in which methane was accounted for 67%, higher than that in the study of Hallaji et al. [30] and Jan Moestedt et al. [36]. The methane concentration varied around 400 mg/L. In order to demonstrate biogas production, it is important to consider the significant parameters with an emphasis on processes which utilized waste such as substrate, VFA concentration, and antibiotic. Additionally, due to the lack of information about biogas production modeling, some efficient ways to solve this issue were addressed [17, 37].

## 4. Conclusions

The pilot-scale anaerobic MBBR system was an appropriate solution for treating piggery wastewater with high OLRs. At OLRs of 10, 15, and 18 kgCOD/m^3^ day and the influent COD of 5.2–5.8 g/L, high COD removal efficiency was obtained in the range of 68–78%.

The first- and second-order kinetics and Monod and Stover–Kincannon models were applied to investigate the organic degradation process. The results found the last model to be the most fit with *R*^2^ 0.98 and the saturation value constant (*K*_*B*_) and maximum utilization rate (*U*_max_) of 52.40 g/L day and 82.65 g/L day, respectively. The first- and second-order kinetics with all 3 HRTs and Monod model with the HRT of 0.56 day also matched with high *R*^2^ values. Therefore, these kinetics and models can be further considered to be used for predicting the kinetic characteristics of the MBBR system in piggery treatment process.

It was found that the yield of biogas and methane production was mostly, respectively, around 0.2 to 0.3 and 0.15–0.20 L/g COD_converted_, respectively. This is because the pilot-scale MBBR system stably operated from day 17 to 80. Some days, the biogas yield reached up to 0.34–0.35 L/g. During this stable operation stage, the cumulative volume of biogas fluctuated from 20 to 30 m^3^/day, reaching approximately 3500 m^3^ during the 17-day digestive process. The methane content in biogas was about 65–70%, and the concentration was around 400 mg/L.

## Figures and Tables

**Table 1:** The characteristic of influent piggery wastewater of the pilot-scale anaerobic MBBR.

Parameters	Ranges (average)	QCVN 01–79 : 2011/BNNPTNT (column B)	QCVN 62 : 2016/BTNMT (column B)
pH	6.6–7.4 (7.05)	5.5–9.0	5.5–9.0
COD (mg/L)	5200–5800 (5482)	100	300
BOD_5_ (mg/L)	2000–3200 (2834)	50	100
TSS	1900–2700 (2326)	100	150
TN (mg/L)	430–630 (548)	30	150
TP (mg/L)	58–80 (67)	6	—

Notes: QCVN01-79 : 2011/BNNPTNT-Annex D1: national technical regulation on sanitary requirement for livestock wastewater; QCVN62 : 2016/BTNMT: national technical regulation on the effluent of livestock; —: not regulated.

**Table 2:** Summary of calculated kinetic coefficients and regression coefficients.

Models	*K*	*K*_*B*_	*U*_max_	*R* ^2^	Conditions
1^st^ order	0.9144/day			0.84	178 days with 3 HTRs: 0.56, 0.37, and 0.3 day
2^nd^ order	0.7459 L/g			0.93	
Monod	2.804/day			0.90	HRT 0.56 day
	3.248/day			0.40	HRT 0.37 day
	2.393/day			0.44	HRT 0.3 day
Stover–Kincannon		52.40 g/L day	82.65 g/L day	0.98	For all 3 HRTs (0.56, 0.37, and 0.3 day), 178-day operation

## Data Availability

The data are all carried out at our laboratories at Faculty of Environmental Sciences, VNU University of Science, 334 Nguyen Trai, Thanh Xuan, Hanoi, Vietnam, and Center for Environmental Monitoring and Modeling, VNU University of Science, 334 Nguyen Trai, Thanh Xuan, Hanoi, Vietnam. The data in the manuscript can be accessed at Faculty of Environmental Sciences, VNU University of Science, 334 Nguyen Trai, Thanh Xuan, Hanoi, Vietnam, and Center for Environmental Monitoring and Modeling, VNU University of Science, 334 Nguyen Trai, Thanh Xuan, Hanoi, Vietnam.
